# Digital intelligence-enabled tumor microbiome research: from mechanistic dissection to clinical translation

**DOI:** 10.3389/fonc.2026.1972980

**Published:** 2026-09-21

**Authors:** Mingliang Shao, Luqi Liu, Qi Wang, Xiaowen Shen, Wei Zhang

**Affiliations:** 1 Department of Interventional Medicine, The Second People’s Hospital of Nantong, Nantong, China; 2 School of Basic Medical Sciences, Jiamusi University, Jiamusi, China; 3 Department of Clinical Laboratory, The Second People’s Hospital of Nantong, Nantong, China

**Keywords:** artificial intelligence, clinical translation, digital intelligence, immunotherapy, machine learning, multi-omics integration, tumor microbiome

## Abstract

Tumor-associated microbiomes are integral components of the tumor microenvironment, modulating tumorigenesis and progression through immune and metabolic pathways. Microbiome data are high-dimensional, sparse, and compositional, which limits conventional analytical approaches. Digital intelligence—an umbrella term encompassing artificial intelligence (AI), machine learning (ML), big-data analytics, and multi-omics integration—has emerged as a powerful paradigm for tumor microbiome research. In this review, we delineate the applications of digital intelligence across the full pipeline: intelligent preprocessing of sequencing data, multi-modal data integration, inference of microbe–host interactions, construction of diagnostic and prognostic models, and discovery of immunotherapy biomarkers. We explicitly distinguish computational association from experimental mechanistic validation and clinical utility, and we critically appraise methodological limitations, including overfitting, data leakage, batch effects, and the scarcity of external prospective validation. Microbiome-related biomarkers are framed as prognostic, predictive, or dynamic, and we caution against equating retrospective performance with clinical value. Finally, we discuss current challenges—data standardization, model interpretability, and ethical privacy—and future directions, including spatial and single-cell microbiomics, FMT-based interventions, and prospectively validated clinical translation.

## 1 Introduction

Tumor-associated microbiomes, residing within tumor tissues, the gut, and other body cavities, modulate tumor biology through immune, metabolic, and inflammatory pathways. Accumulating evidence demonstrates that intratumoral microbiomes regulate anti-tumor immunity and T-cell metabolism. For instance, the abundance of *Staphylococcus* within breast tumors correlates positively with CD8^+^ T-cell activation and suppresses triple-negative breast cancer (TNBC) growth via NAD metabolite consumption (1). In lung cancer, alterations of the gut microbiome promote tumor growth and dampen immunotherapy responses through IL-6-dependent mechanisms (2). Beyond these examples, the tumor microbiome comprises tumor-type-specific intracellular bacterial communities that differ across cancer types (3), and it shapes innate immune cell function, thereby influencing the initiation and magnitude of anti-tumor immune responses and playing pivotal roles in tumor immune evasion (4). Collectively, these findings establish the microbiome as an indispensable constituent of the tumor microenvironment.

Nevertheless, microbiome data are characterized by high dimensionality, sparsity, and compositionality (5, 6). Variations in sequencing depth and sample processing introduce abundant zero values and technical noise, while the compositional nature of relative abundances renders inter-species dependencies intractable for traditional statistical methods (5, 6). In recent years, digital intelligence—an umbrella term that we use here to encompass AI, ML, big-data analytics, and multi-omics integration as applied to tumor microbiome research—has provided powerful tools for the field, enabling end-to-end empowerment from data acquisition and feature extraction to model prediction (7). ML algorithms, particularly adept at handling high-dimensional complex data, have been widely applied in disease diagnosis, prognostic prediction, and biomarker discovery (8). Multi-omics integration has further unveiled synergistic interactions among microbiomes, host genomes, transcriptomes, and metabolomes (9, 10). Intelligent diagnostic and prognostic models based on microbiome signatures have demonstrated promising performance, as reviewed across cancer types (7) and illustrated for melanoma (11). Throughout this review, we distinguish computational associations from experimentally validated mechanisms and from clinical utility, and we caution that retrospective model performance must not be equated with clinical value. This review summarizes the technical framework, current applications, challenges, and future directions of digital intelligence in tumor microbiome research (**Figure 1**).

**Figure 1:**
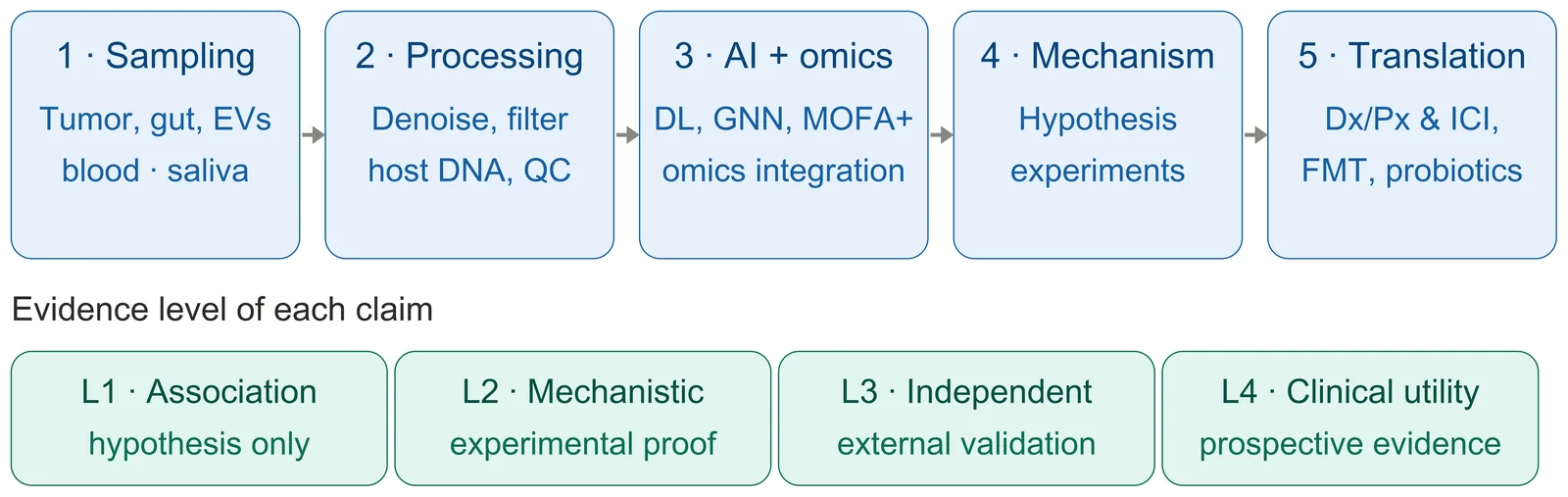
Digital-intelligence-enabled tumor microbiome research: from sampling to clinical translation. The workflow spans five stages: (1) sample acquisition (tumor tissue, gut, saliva, blood, extracellular vesicles); (2) intelligent preprocessing of high-throughput sequencing data (host DNA filtration, denoising, chimera removal, quality control); (3) AI/ML and multi-omics integration (deep-learning taxonomy, network inference, graph neural network fusion, multi-omics factor analysis); (4) mechanistic dissection and experimental validation of microbe–host interactions; and (5) biomarker development and clinical translation (diagnostic/prognostic models, ICI-response prediction, FMT and engineered probiotics). The lower panel defines the evidence level assigned to each claim throughout the review: L1, computational association/hypothesis generation; L2, experimental mechanistic validation; L3, independent (external) clinical validation; L4, demonstrated clinical utility based on prospective evidence. Cross-cutting challenges—data standardization, model interpretability, and ethical privacy—are indicated by the dashed frame.

## 2 Intelligent preprocessing of high-throughput sequencing data

High-throughput sequencing, including metagenomics, 16S rRNA, and transcriptome sequencing, constitutes the primary data source for tumor microbiome studies. Raw data are often contaminated by host DNA, sequencing errors, and low-quality reads. Standard bioinformatics pipelines therefore perform error correction and chimera removal before downstream analysis: for amplicon data, error-model-based denoising (e.g., DADA2) distinguishes biological sequence variants from sequencing noise (12), and chimera filtering (e.g., UCHIME) removes artifacts generated during PCR amplification (13). In parallel, deep learning has been applied to taxonomy assignment itself: DeepMicrobes, for example, uses deep neural networks to classify metagenomic reads with high accuracy, complementing alignment-based classifiers (14). For metagenomic assembly, integrated deep learning approaches that incorporate sequence co-abundance and tetranucleotide frequency information construct denoised latent spaces, enabling precise genome binning and reconstruction of more near-complete genomes than conventional methods (15). Automated quality control pipelines, combining tools such as FastQC with AI-driven anomaly detection algorithms, facilitate rapid identification and exclusion of low-quality data, improving standardization and reproducibility (5).

## 3 Multi-modal data integration strategies

Tumor microbiome research is increasingly multi-modal, integrating clinical information, imaging data, and diverse omics datasets. The heterogeneity in data sources, formats, and structures poses a central challenge for revealing complex microbe–host interactions. Graph neural networks (GNNs) provide a flexible framework for fusing heterogeneous data, and scalable training algorithms—such as GraphSAINT, a general graph sampling-based inductive learning method—enable GNN-based fusion models to be trained on large biomedical graphs (16). Knowledge graph technologies, such as MicrobiomeKG, extract knowledge assertions from the literature to construct networks that support hypothesis generation and integrative analysis (17). Additionally, multi-omics factor analysis models such as MOFA+ integrate metagenomic and metabolomic data, leveraging phylogenetic tree structures to aggregate rare features and elucidate how microbiomes influence gut metabolite abundances (9). These digital integration strategies lay the essential data foundation for subsequent mechanistic dissection and clinical translation.

## 4 Dissection of microbe–host interaction mechanisms

Microbiomes engage in complex “crosstalk” with host cells via metabolites and surface molecules, regulating immune responses and signal transduction pathways. Given the extreme complexity of these interaction networks, traditional experimental approaches alone cannot comprehensively identify key nodes and regulatory mechanisms. Machine learning-based network inference algorithms provide powerful tools for generating hypotheses about microbe–host interactions from large-scale metagenomic datasets. For example, SPIEC-EASI infers sparse microbial ecological networks from compositional abundance data, revealing co-occurrence patterns among taxa (18), whereas MIMOSA uses metabolic models to predict links between microbial taxa and metabolite concentrations (19). It is important to emphasize that such computational methods identify associations and generate hypotheses; they do not, by themselves, establish biological mechanisms, which require experimental validation.

A representative example is the *Fusobacterium nucleatum*–Wnt/β-catenin axis in colorectal cancer (CRC). Random forest models integrating metagenomic and transcriptomic data identified a computational association between *F. nucleatum* abundance and Wnt/β-catenin pathway activation; subsequent independent experiments confirmed that *F. nucleatum* promotes CRC cell proliferation and tumor progression by binding host E-cadherin via its surface adhesin FadA, thereby activating β-catenin signaling (20). Similarly, in hepatocellular carcinoma, deoxycholic acid produced by specific gut microbiota was shown in experimental studies to activate TLR4 signaling and drive tumor progression (21). These examples illustrate the bridging role of digital intelligence from associative analysis to mechanistic validation, provided that computational predictions are confirmed experimentally before translational claims are made.

## 5 Construction of tumor diagnostic and prognostic models

Intelligent diagnostic models based on microbiome signatures have demonstrated substantial clinical potential across multiple cancers. In CRC, machine learning models such as XGBoost and random forest, trained on fecal microbiome data, exhibit excellent predictive performance with area under the curve (AUC) values around 0.90 (22, 23). Zackular et al. (22) significantly improved adenoma–carcinoma discrimination by integrating gut microbiome data with clinical risk factors. In pancreatic cancer, a machine learning model utilizing serum extracellular vesicle microbiome markers achieved a test AUC as high as 0.96 (24). For prognostic prediction, microbiome abundance-based prognostic scoring models in breast cancer can independently predict overall survival regardless of traditional clinical factors, with specific microbial signatures significantly associated with tumor stage and recurrence risk (25). Long short-term memory (LSTM) networks and other recurrent neural network models excel at processing temporal data (26), and multi-task learning models simultaneously predict prognosis and recurrence risk by sharing underlying feature representations, thereby enhancing predictive accuracy (27). Combining microbial biomarkers with clinical features such as carcinoembryonic antigen further improves diagnostic performance, with AUC values reaching 0.94 in cross-cohort analyses (28).

Although these results are encouraging, we emphasize that high retrospective AUC values reflect model discrimination within the analyzed cohorts and should not be equated with clinical utility. Robust evaluation requires nested cross-validation, external validation in independent cohorts, and, ultimately, prospective studies with decision-analytic assessment of net benefit (see also Section 8). **Table 1** summarizes representative studies across cancer types, including their computational methods, validation strategies, key findings, and major limitations.

**Table 1:** Representative studies of AI/multi-omics-enabled tumor microbiome analysis.

Cancer type	Sample type	Computational method	Clinical application	Validation strategy	Key findings	Major limitations
Colorectal cancer	Fecal metagenomics	XGBoost/random forest	Early diagnosis	Independent cohort/cross-validation	Adenoma–carcinoma discrimination, AUC ≈ 0.90 (22, 23)	Retrospective; cohort heterogeneity; batch effects
Colorectal cancer (cross-cohort)	Fecal metagenomics	Cross-cohort meta-analysis	Diagnosis	Multi-center external cohorts	Cross-cohort microbial signature; AUC 0.94 combined with CEA (28)	Prospective validation required
Pancreatic cancer	Serum extracellular vesicles	Machine learning	Diagnosis	Test set	Test AUC 0.96 (24)	Small sample; single center
Breast cancer	Tumor tissue 16S	Prognostic scoring model	Prognosis	Independent cohort	Microbiome signature independently predicts overall survival (25)	Retrospective
Breast cancer (TNBC)	Intratumoral bacteria + metabolome	16S + mass spectrometry + functional assays	Mechanism/potential biomarker	Two independent cohorts + mouse models	*Staphylococcus* depletes NAD, activates CD8^+^ T cells, suppresses tumor growth (1)	Low-biomass contamination risk
Lung adenocarcinoma	Gut microbiome (models + patients)	FMT + single-cell/flow cytometry	Mechanism (IL-6 axis)	Animal + patient validation	*Alistipes* expansion → gut inflammation → IL-6 → immunosuppression, reduced ICI efficacy (2)	Predominantly preclinical
Melanoma	Fecal metagenomics	Cross-cancer multi-cohort association	ICI-response prediction	Multi-cohort	*F. prausnitzii*/*B. adolescentis* associated with response (31)	Association, not causation; no comparator arm
Melanoma	Fecal	FMT (clinical intervention)	Overcoming anti-PD-1 resistance	Phase I/II trials	FMT + anti-PD-1 enhanced response (36, 37)	Single center; small cohorts
NSCLC/melanoma	Fecal	FMT + ICI (phase 2 trial)	First-line therapy	Multicenter phase 2 (FMT-LUMINate)	ORR 80% (NSCLC), 75% (melanoma); benefit linked to depletion of deleterious taxa (38)	Single-arm; n = 40; randomized confirmation needed
Oral squamous cell carcinoma/CRC	Tissue spatial transcriptomics	10x Visium + single-cell RNA-seq (INVADEseq)	Mechanism (spatial heterogeneity)	Patient tissues + functional studies	Bacteria populate immunosuppressive microniches (52)	Emerging technology; low throughput

## 6 Discovery of immunotherapy biomarkers and intervention strategies

Gut microbiome composition is closely linked to the efficacy of immune checkpoint inhibitors (ICIs). By modulating the composition and function of the tumor immune microenvironment, specific microbial abundances serve as potential biomarkers for predicting ICI responses (29, 30). Lin et al. (31), through cross-cancer multi-cohort analysis, demonstrated that the abundances of *Faecalibacterium prausnitzii* and *Bifidobacterium adolescentis* positively correlate with immunotherapy response, identifying these species as stable biomarkers across studies. Recent studies have refined the cancer-specific landscape: *Akkermansia muciniphila* predicts clinical response to PD-1 blockade in advanced non-small cell lung cancer (NSCLC) (32), and cross-cohort, strain-level gut microbiome signatures are associated with ICI response in advanced melanoma (33). Dietary fiber and probiotic intake further shape the gut microbiome and may influence melanoma immunotherapy outcomes (34).

A rigorous translational framework requires distinguishing biomarker types. *Prognostic* biomarkers are associated with outcome regardless of treatment; *predictive* biomarkers interact with treatment and can only be established through comparator-arm or randomized designs; *dynamic (pharmacodynamic)* biomarkers reflect on-treatment changes, such as longitudinal microbiome shifts during ICI or FMT (11, 35). Accordingly, associations between baseline microbiome composition and outcome within ICI-treated cohorts should, in most cases, be interpreted as prognostic or treatment-context associations rather than treatment-specific predictive biomarkers. Major confounders—including prior or concomitant antibiotic use, diet, chemotherapy or corticosteroids, tumor stage and histology, PD-L1 status, and metformin—must be controlled in study design and analysis (32, 33). AI models that integrate metagenomic data with clinical outcomes can quantify the predictive value of microbial biomarkers and construct prediction models, but their outputs require external and prospective validation before clinical use (7, 11, 35).

Regarding intervention strategies, fecal microbiota transplantation (FMT) has been shown to overcome resistance to anti-PD-1 therapy in patients with advanced melanoma (36, 37). More recently, the phase 2 FMT-LUMINate trial demonstrated clinical activity of healthy-donor FMT combined with anti-PD-1 in first-line NSCLC (objective response rate 80%) and with dual anti-PD-1/anti-CTLA-4 blockade in melanoma (75%); longitudinal metagenomic monitoring revealed that clinical benefit was associated with depletion of deleterious baseline taxa rather than donor strain engraftment (38). These findings support ongoing randomized evaluation of FMT-based strategies (38). The convergence of synthetic biology and AI offers new possibilities for designing engineered bacterial strains for tumor-localized delivery of therapeutic payloads (39), while adaptive dosing strategies informed by real-time microbiome monitoring are under exploration (7). Notably, the gut–immune axis is increasingly recognized as a bidirectional system whose disruption underlies multiple immune-related diseases, underscoring the need for mechanism-based biomarker development (40).

## 7 Data standardization and quality control

The reproducibility of microbiome research is significantly affected by technical factors spanning sample collection, processing, and sequencing. Discrepancies in metadata collection, bioinformatics pipelines, and statistical methodologies across studies compromise the reproducibility of AI models across datasets (41). In low-biomass samples, reagent contamination is particularly pronounced and can introduce false-positive results (42). Establishing standardized operating procedures and cross-platform normalization algorithms is therefore imperative; statistical methods such as ComBat-seq can effectively correct batch effects in count data (43). Furthermore, standardized protocols for human sample processing and the establishment of global microbiome reference databases are essential for evaluating and calibrating laboratory-specific workflows (44).

## 8 Model interpretability and clinical translation bottlenecks

The “black-box” nature of deep learning models severely constrains clinical trust. Although models may identify microbial features associated with immunotherapy response, they cannot, on their own, elucidate the underlying metabolic or immunomodulatory mechanisms (45). Explainable AI techniques such as SHAP and LIME are employed to reveal the microbial features contributing to model predictions (46); however, computational complexity increases substantially when processing large-scale high-dimensional data, and these methods explain model behavior rather than underlying biology (45). More critically, most AI models remain confined to retrospective studies, lacking rigorous validation in independent prospective cohorts. Clinical translation requires regulatory approval and workflow integration, and reporting guidelines such as DECIDE-AI have been developed to standardize the early-stage clinical evaluation of AI-based decision-support systems (47).

Several methodological limitations warrant explicit attention (7, 45, 47). First, microbiome datasets are typically high-dimensional with modest sample sizes, rendering models prone to overfitting; nested cross-validation and feature-stability analyses are therefore essential. Second, data leakage—including sample overlap between training and test cohorts and temporal leakage in longitudinal or ICI-response models—can inflate performance estimates and must be explicitly guarded against. Third, batch effects arising from differences in DNA extraction, sequencing platforms, and bioinformatics pipelines can dominate biological signal; harmonization (e.g., ComBat-seq (43)) and per-batch validation are required. Fourth, high performance in the discovery cohort does not guarantee generalizability across populations, platforms, or cancer types; independent external validation and prospective evaluation are prerequisites for clinical adoption. Bridging the “translation gap” from algorithmic accuracy to clinical trust necessitates a shift from model-centric optimization to system-level accountability, emphasizing cross-institutional validation and interpretability-by-design (45, 47).

## 9 Ethical privacy and data security

Microbiome data contain rich individual health information, rendering multi-center data sharing vulnerable to privacy breaches. Even after de-identification, the possibility of re-identifying individuals through microbiome characteristics has been demonstrated (48). Privacy-preserving technologies such as differential privacy and homomorphic encryption have been proposed, albeit at the cost of significantly increased computational overhead and potential model performance degradation (49). Existing ethical and legal frameworks for medical data (50, 51) predominantly target traditional genomic data and inadequately address the unique attributes of microbiome data. Updated ethical guidelines and specialized regulatory frameworks for microbiome data sharing and usage are urgently needed (50, 51).

## 10 Trends in frontier technology integration

Digital intelligence-enabled tumor microbiome research is advancing toward multi-technology integration. The combination of single-cell microbiomics and spatial transcriptomics promises to reveal microbial heterogeneity and spatial distribution within the tumor microenvironment, directly linking microbial localization with host cell transcriptional states; landmark studies using spatial transcriptomics and single-cell RNA sequencing have shown that intratumoral bacteria populate immunosuppressive microniches and interact with specific host cells (52). Quantum computing, with its powerful parallel processing capabilities, may eventually optimize computationally intensive tasks such as large-scale metabolic network simulation and drug screening, although practical applications remain at an early, speculative stage (53). Digital twin technology—which integrates patient microbiome, genomic, and clinical data to construct virtual models capable of simulating the long-term impact of different interventions—offers a conceptual framework for “trial-before-treatment” precision medicine; however, its implementation requires robust data infrastructure and validation (54).

## 11 Conclusion

Digital intelligence technologies are propelling tumor microbiome research into a new paradigm. From data acquisition and mechanistic dissection to clinical translation, AI and multi-omics integration have become core engines driving the field forward. Deep learning-based microbial biomarker screening and diagnostic models have demonstrated promising performance across multiple malignancies, opening new avenues for liquid biopsy and early screening. In immunotherapy, digital intelligence has facilitated the identification of microbiome features associated with ICI efficacy and the development of microbiome-targeted intervention strategies, including FMT-based approaches now entering randomized evaluation. Nevertheless, challenges in data standardization, model interpretability, and ethical privacy require interdisciplinary collaboration. Looking ahead, the integration of frontier technologies—including single-cell and spatial microbiomics and, speculatively, quantum computing and digital twins—will help move tumor microbiome research from correlative analysis toward causal mechanistic validation and precision intervention. This progression will require rigorous experimental validation and independent prospective clinical evaluation to translate precision oncology from concept to practice.
